# Comorbidities and Cofactors of Anaphylaxis in Patients with Moderate to Severe Anaphylaxis. Analysis of Data from the Anaphylaxis Registry for West Pomerania Province, Poland

**DOI:** 10.3390/ijerph18010333

**Published:** 2021-01-05

**Authors:** Iwona Poziomkowska-Gęsicka, Magdalena Kostrzewska, Michał Kurek

**Affiliations:** 1 Clinical Allergology Department, Pomeranian Medical University (PMU) in Szczecin, 70-204 Szczecin, Poland; alergologia@spsk2-szczecin.pl; 2 Department of Pulmonology, Allergology and Respiratory Oncology, University of Medical Sciences, 60-569 Poznan, Poland; m0304@tlen.pl

**Keywords:** anaphylaxis, epidemiology, cofactors, comorbidity

## Abstract

Anaphylaxis is a severe, potentially life-threatening systemic hypersensitivity reaction that is still rarely diagnosed. For safety reasons, patients should visit an allergologist to identify potential causes and cofactors of this reaction. This paper presents the analysis of data from the Anaphylaxis Registry gathered over ten years at the Allergy Clinic, Pomeranian Medical University (PMU). A questionnaire-based survey was used for patients visiting the Allergy Clinic to identify potential augmentation factors/comorbidities and/or cofactors of anaphylaxis in patients with a history of moderate to severe anaphylaxis. The registry comprised patients with grade II or higher anaphylaxis. The gathered data concerned chronic comorbidities (cardiovascular diseases, respiratory diseases, and others), recurrence of anaphylaxis, and potential cofactors in anaphylaxis. In the analyzed group, the incidence rate of anaphylaxis was the highest for women aged 19–60 years. Most common comorbidities in patients with moderate to severe anaphylaxis included: cardiovascular diseases, respiratory tract diseases, features of atopy, and thyroid diseases. More than 30% of drug-induced reactions were anaphylactic reactions due to the re-exposure to the same drug, which points to the need for educational initiatives in this area. The incidence rate of anaphylaxis induced by Hymenoptera stings was comparable in patients who had a previous generalized reaction and those who had good tolerance to the previous sting. It is important to take these cofactors into consideration when evaluating patients with anaphylaxis as they may play a role in future anaphylactic reactions.

## 1. Introduction

The term *anaphylaxis* was used for the first time in 1902 by Portier and Richet to describe a reaction opposite to prophylaxis. They described an experiment on dogs that had tolerated a certain dose of a jellyfish toxin, but after the injection of a lower dose of the same toxin, the dogs reacted with bronchospasm and cardiorespiratory arrest and died [1].

Different definitions of anaphylaxis have been proposed since the first use of this term—Table 1. Contemporary definitions are presented in a paper by Turner in 2019 [2].

Generally, anaphylaxis is most commonly defined as an acute, severe, potentially life-threatening systemic hypersensitivity reaction [4] and remains a clinical diagnosis. There are also definitions without the word ‘acute’ or without the adjective ‘allergic’ reaction. Anaphylaxis may also be delayed, with the onset 4–6 h after the intake of food, or without the involvement of immunologic mechanisms. It should be kept in mind that the onset of anaphylaxis after stings or allergen injections is usually rapid; 70% begin in less than 20 min, and 90% in less than 40 min [7]. Most healthcare professionals define anaphylaxis as a serious, generalized, allergic, or hypersensitivity reaction that can be life-threatening and even fatal [8,9,10,11,12].

Depending on the pathomechanism, anaphylactic reactions are classified as allergic anaphylaxis (usually IgE-dependent), non-allergic anaphylaxis, and the so-called cytokine storm with the involvement of new G-coupled receptor MRGPRX2, located on mast cells [13]. In the literature, there are several classification systems, considering the severity of anaphylaxis and clinical symptoms, and proposed, for example, by Ring and Messmer [14], Muller [15], Brown [16], Muraro [17], and Mehl [18]. Table 2 below presents the classification of anaphylaxis severity by Ring and Messmer (adopted in this article).

Many publications, apart from the analysis of causes of anaphylaxis, present information about patient-specific risk factors and cofactors amplifying anaphylactic reaction [19,20,21,22,23,24,25,26,27]. Multiple episodes of anaphylaxis following the consumption of unconnected foods should raise concerns about the possibility of a hidden allergen-induced or “summation anaphylaxis” due to cofactor influence [28]. Skypala provides an overview of hidden allergens and the influence of cofactors in food-related anaphylaxis. An accurate clinical history with a high index of suspicion is paramount in making a correct diagnosis [29].

Severe anaphylaxis is associated with older age, asthma, and chronic obstructive pulmonary disease (COPD), and pharmacotherapy [30]. According to different authors and reports, the role of cofactors in about 30% of anaphylactic reactions has been documented, from 25.6% in France to 39% in Germany [31]. Cofactors, including exercise, ethanol, acute infections, and stress potentially amplify anaphylaxis by decreasing the threshold of allergen exposure (the allergen “dose”) needed to trigger anaphylaxis in patients with low or borderline allergen sensitization [19,32,33,34]. An analysis of data from the European Anaphylaxis Registry assessed factors increasing the risk for a severe anaphylactic reaction [35]. The following augmentation factors or cofactors were listed by Worm [35]:

1. Non-modifiable/stable/independent/intrinsic
2. Modifiable/unstable/dependent/extrinsic

Nonmodifiable factors include age, sex, comorbidities, basic tryptase level, and a previous reaction triggered by the same factor.

Modifiable factors include long-term pharmacotherapy, especially with non-steroidal anti-inflammatory drugs (NSAIDs), proton pump inhibitors (PPIs) [31], exercise, alcohol, and emotional stress.

Acute infections, especially the early phase of infection, during specific immunotherapy (SIT) and food immunotherapy, are cofactors in anaphylaxis. It has been assumed that bacterial or viral products can be sensed by receptors on mast cells and basophils and, under certain conditions, trigger or enhance mast cell degranulation [31].

Old age, combined with comorbidities such as cardiovascular disease (CVD) and asthma, especially allergic asthma, is an important risk factor for severe anaphylaxis with hospitalization, prolonged hospital stay, and fatality [20,21,23,36,37]. There are also reports on studies in a group of patients who have unexplained recurrent episodes of severe anaphylaxis with CVD and elevated basal tryptase levels (>11.4 mcg/L) [19,26].

According to the literature on anesthesiology, factors that enhance the risk of anaphylaxis include old age, female sex, lactation, asthma, fever, systemic mastocytosis, active infection, spinal anesthesia, pre-menstrual state, and emotional state [38].

A review published in 2018 referenced many other cofactors, including menstruation, infection, extreme air temperatures, cannabis use, and medications other than NSAIDs, including angiotensin-converting enzyme inhibitors, beta-blockers, and antacids [39].

Certain factors place some individuals at increased risk for more severe anaphylactic reactions: (1) history of an anaphylactic reaction; (2) history of asthma, especially if poorly controlled; (3) allergy to peanuts, nuts, fish, and shellfish; (4) teenage patients, and 5) patients on β-blockers or angiotensin-converting enzyme inhibitors [40].

The most common cause of anaphylaxis mentioned in Polish and German registries are Hymenoptera stings [41,42]. Current German and European guidelines recommend Venom Immunotherapy (VIT) for all patients with grade II or higher reactions and for patients with a grade 1 reaction if they have any other risk factors or if their quality of life has been negatively impacted [43,44,45,46]. Other risk factors for severe anaphylaxis triggered by insect sting include male sex, older age, a large number of stings, a short time interval between stings, the location of the sting, the absence of skin symptoms, high baseline serum tryptase levels, as well as cardiovascular comorbidity [47]. Oropeza et al. [48] also reported other risk factors, such as asthma, rhinitis, atopic dermatitis, urticaria, and/or angioedema.

The presence of cofactors is associated with a more severe anaphylactic reaction and reduces the amount of the allergen needed to trigger anaphylaxis. Reports from the United States emphasize the role of education on anaphylaxis, as the majority of patients experience subsequent episodes of anaphylaxis [7].

## 2. Objective

The objective of this study was to identify factors increasing the severity of anaphylaxis/cofactors/comorbidities in patients with a history of moderate to severe anaphylactic reactions.

## 3. Material and Methods

### 3.1. Study Design and Data Collection

In the retrospective analysis, we used a questionnaire-based survey completed by doctors specialized in allergology. Details on the study design are presented in another publication [42]. Of all 10,738 new patients examined at the Allergology Department in 2006–2015, with suspicion of any allergy or non-allergic hypersensitivity, we found above 490 patients with suspicion of moderate and severe anaphylaxis. One year after the first visit, each was analyzed again using the survey and additional results were collected. The study protocol did not require the approval of a Bioethics Committee.

The basic questionnaire was a simplified version of the Network for Online-Registration of Anaphylaxis survey (NORA) from Berlin [42]; more information in the Appendix A.

Patients were asked about their sex and age at the time of the anaphylactic episode, as well as about:

Presence of chronic diseases affecting the cardiovascular system, lower respiratory tract, upper respiratory tract, thyroid, gastrointestinal tract, kidneys, as well as diabetes, features of atopy, and others. Definition of atopy by Johansson: “Atopy *is* a personal and/or familial tendency, usually in childhood or adolescence, to become sensitized and produce IgE antibodies in response to ordinary exposures to allergens, usually proteins. As a consequence, these persons can develop typical symptoms of asthma, rhinoconjunctivitis, or eczema.” At the first visit, the patients indicated whether they suspected or had a confirmed atopic disease (phenotypes listed above); later, one year after reporting, an allergist specialist either confirmed or removed the existence of atopic features (atopic disease, e.g., the reported allergic rhinitis (AR) turned out to be chronic non-allergic rhinitis). Other aspects were defined as follows:

Recurrence of anaphylaxis, i.e., re-exposure to the same trigger or a pharmaceutical with a similar chemical structure or activity, after the initial reaction.

Additional factors associated with anaphylaxis, e.g., alcohol intake, exercise, symptoms of acute infection, and menstruation.

### 3.2. Statistical Analysis

Obtained data were analyzed using the Statistica 12 software package (StatSoft, Inc., Cracow, Poland license, Tulsa, OK, USA). A basic statistics panel was used for data processing: for descriptive statistics and qualitative variables used in the analysis, non-parametric tests were used. The collected data were presented in the form of a multi-division table. For qualitative variables, we used Pearson’s chi-square test, the Wilcoxon non-parametric signed-rank test, other significance tests, and structural indicators. Statistical significance was adopted at a *p*-value of *p* < 0.05.

## 4. Results

### 4.1. Demographic Data

Of all 10,738 new patients examined at the Allergology Department in 2006–2015, there were 382 cases of moderate and severe anaphylaxis (grades II-IV by Ring and Messmer classification), which accounted for 3.56% of new patients.

**Figure 1:**
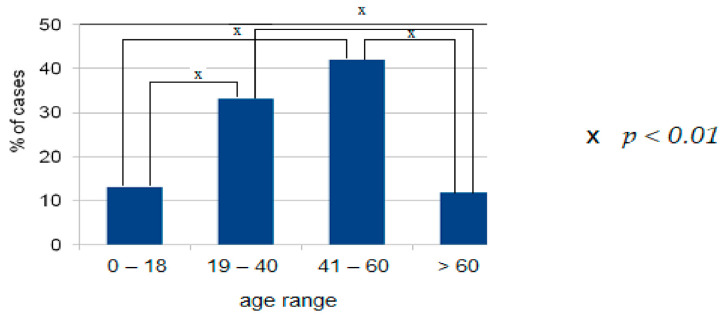
Distribution of patients by age range in the analyzed group.

There were 236 women (61.8%) and 146 men (38.2%) with anaphylaxis, *p* < 0.001. In This group was 50 children (13.1%): 29 girls (7.6%), 21 boys (5.5%). Adults vs. children *p* < 0.001. The mean age at the onset of anaphylaxis was 40.4 years (range 0–79) for women (*n* = 236), and 38.2 years (range 0–76) for men (*n* = 146). In the group of children and adolescents (max. age 18 years), the mean age at onset was 11 years (9.9 for boys and 12.1 for girls). Most patients with moderate to severe anaphylaxis were in the age range 19–40 and 41–60 years, which accounted for about 75% of all anaphylactic reactions in the registry. A detailed distribution of data for the analyzed group is presented in Figure 1.

**Figure 2:**
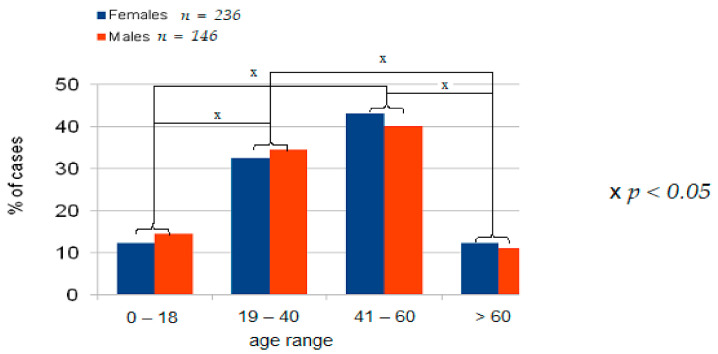
Distribution of patients by age range and sex in the analyzed group.

There were no significant differences in the proportion of male and female patients within age groups: females vs. males Figure 2, but there were significant differences in the proportion of males and females between age groups.

### 4.2. Comorbidities 

The most common comorbidities recorded in patients with moderate to severe anaphylaxis include cardiovascular disease, upper respiratory tract disease (mainly allergic rhinitis), lower respiratory tract diseases (mainly asthma), features of atopy, thyroid disease, diabetes mellitus (mainly type 2).

**Figure 3:**
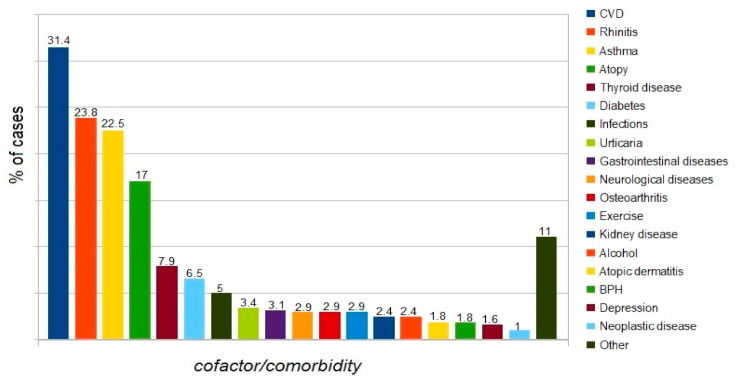
Proportion of patients with a specific cofactor/comorbidity in the analyzed group.

Data on comorbidities in the analyzed group are presented in Figure 3. Note, one patient may have more than one cofactor and or comorbidity.

**Figure 4:**
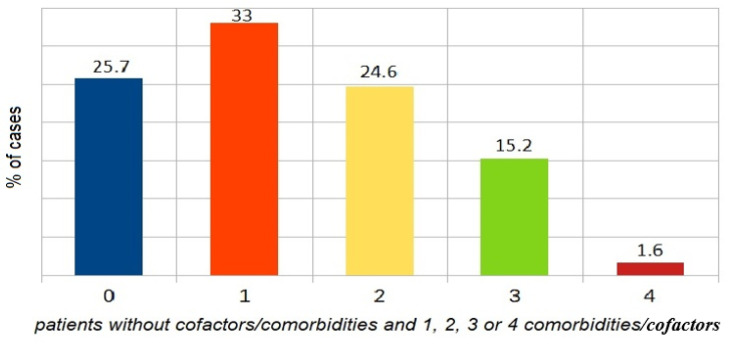
The proportion of patients without cofactors/comorbidities and those with one, two, three, or four comorbidities/cofactors.

Presence of comorbidity/cofactor: one factor vs. two vs. three vs. four vs. zero is shown in Figure 4. The comparison focused on the presence of one vs. two vs. three vs. four vs. zero comorbidity/cofactor revealed a significant difference between patients with one and those with three comorbidities/cofactors (*p* < 0.01). Anaphylaxis was significantly more frequent in patients with one cofactor. No differences were found between patients with four comorbidities vs. other variants because of the low number of patients with four comorbidities.

**Figure 5:**
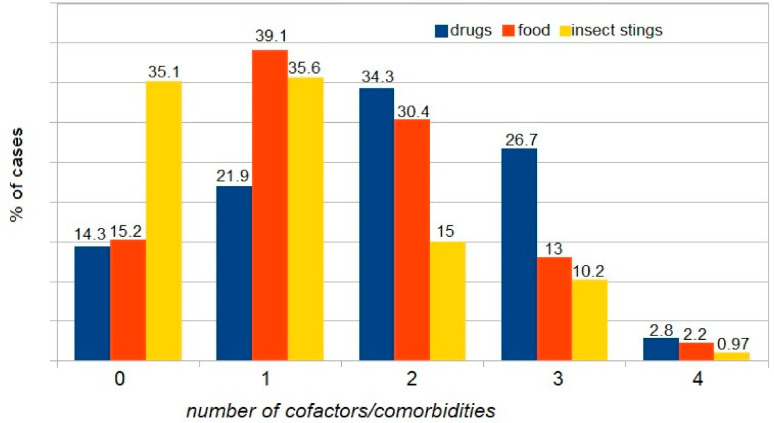
The proportion of patients without cofactors, vs. one, vs. two, vs. three, and vs. four cofactors, with triggers of anaphylaxis considered.

**Figure 6:**
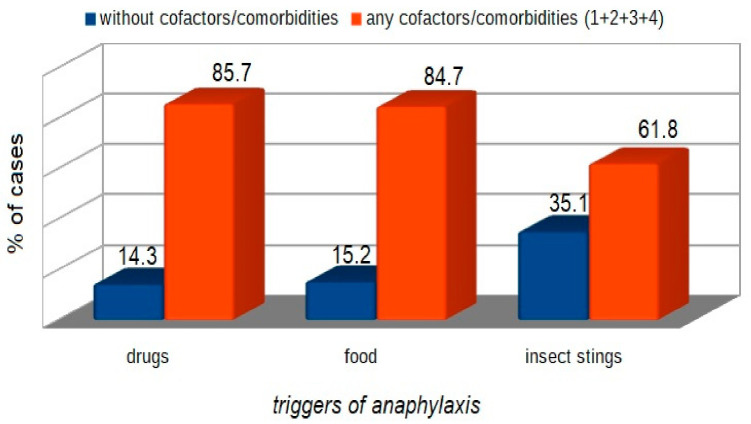
Comparison of patient subgroups depending on the trigger of anaphylaxis and presence or absence of comorbidities (insect stings *p* < 0.001, food *p* < 0.001, drugs *p* < 0.001).

Comorbidities versus triggers of anaphylaxis (insect stings, drugs, food) are presented in Figure 5. The incidence of anaphylaxis was significantly higher in patients with comorbidities compared to those without comorbidities (Figure 6).

### 4.3. Tryptase 

Elevated basal levels of tryptase in blood were found only in 15/148 patients (10.14%). However, not all patients had their tryptase level measured. This test was done only in 148 out of 382 patients (38.74%) with moderate to severe anaphylaxis; details shown in Table 3.

### 4.4. Drugs

**Figure 7:**
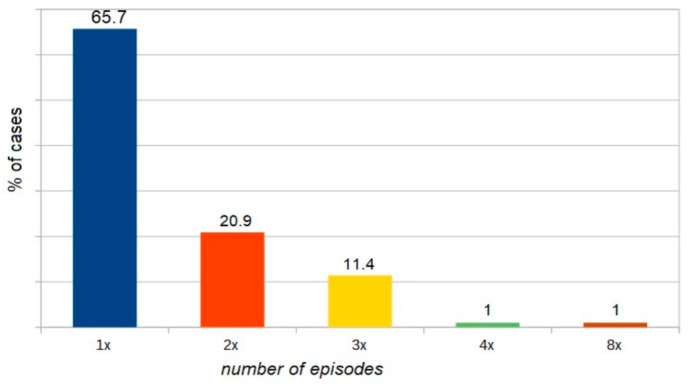
The proportion of patients with drug-induced anaphylaxis versus the number of episodes (one vs. two vs. three vs. four vs. eight).

Almost 66% of moderate to severe anaphylactic reactions developed after the first exposure to a drug, or in patients who had previously showed good tolerance to a given drug. However, 34% of anaphylactic reactions to drugs were recorded in patients who had a history of at least one anaphylactic reaction to the same drug or a drug from the same chemical class (Figure 7, one episode vs. more than one episode; *p* < 0.005).

**Figure 8:**
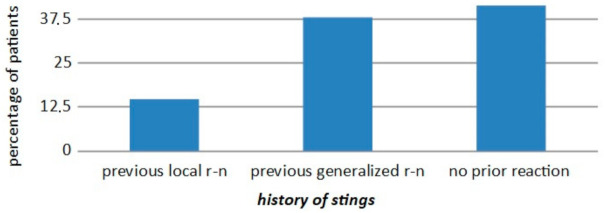
The proportion of patients with insect sting anaphylaxis versus those with a history of stings with previous local reaction (r-n) or previous generalized reaction, or no prior reaction (tolerance).

Insect stings—statistics on the history of local vs. systemic reactions (*p* < 0.001) are presented in Figure 8. 

Exercise as a cofactor—11 patients (including five with Food-dependent exercise-induced anaphylaxis (FDEIA)). In this group of patients, the exercise challenge test was negative. FDEIA was diagnosed using molecular tests—Table 4 (these cases were previously labeled as idiopathic anaphylaxis).

## 5. Analysis of Results and Discussion

In our study, the incidence of moderate to severe anaphylaxis in the group of patients referred for all causes to an allergy specialist was estimated at 0.35% per year. The annual incidence rate for the West Pomerania province was 2.3/100,000, which is close to the lower limit reported by Wolbing (3.2–68.4) [31]. This is also consistent with data reported by Panesar et al. for the European population [4], where the incidence rate ranged from 1.5 to 7.9 per 100,000 person-years. In 2015, the prevalence rate of anaphylaxis in Poland (according to the National Health Fund of Poland, NHF) was much higher, but differed considerably between regions and was estimated at 8.2 per 100,000 [49], and a similar rate was reported by Tejedor-Alonso et al. [50]. In the general population of the United States, the incidence rate was estimated at 1.6% [7].

Sex and age: Patients aged 19–60 years accounted for 75% of all anaphylaxis cases in the analyzed registry. Anaphylaxis was more frequent in females than in males (*p* < 0.001), which is explained by the promoting effect of estrogens in anaphylaxis. Similar data were reported by other researchers, who indicated the reproductive age of women as an augmenting factor in anaphylaxis [13]. Estrogen might also play a role by enhancing endothelial expression of nitric oxide synthase and nitric oxide production, increasing vascular permeability, and intensifying anaphylaxis severity [19,33]. In the analyzed group, we found no increased incidence of anaphylaxis among adolescents and patients older than 60 years, unlike Muñoz-Cano et al. [13], who emphasized the association between old age and anaphylaxis due to comorbidities and increased use of medications. Increased incidence of anaphylaxis in adolescents has been attributed to their “risky behavior” [13,51]. Data from the European and Korean registries suggest that the risk factors for severe anaphylaxis include older age [35] and male sex [21], but this was not supported by findings from our analysis. Ruëff et al. [52] and Chen et al. [53] investigated insect venom-allergic patients and indicated male sex as a risk factor for severe anaphylaxis. Older age [54] increases the risk of severe anaphylaxis, but in the presented material, the rate of anaphylaxis in patients older than 60 years was similar to that in the age range 0–18 years and significantly lower than in age ranges 19–40 (*p* < 0.005) or 41–60 years (*p* < 0.001).

Tryptase—basal level (not at the time of anaphylactic reaction): In the analyzed group, the rate of patients with elevated (>11.4 µg/L) tryptase levels (not always with diagnosed mastocytosis) was higher than 10%, vs. 1.64% of subjects with diagnosed mastocytosis according to other anaphylaxis registers [35]. The highest percentage of patients (>6%) with elevated tryptase levels has been observed in the age range 41–60 and in the group of patients aged more than 60. According to Kucharewicz, in the group of patients with Hymenoptera sting anaphylaxis, similar to our study, the rate of patients with elevated tryptase level relative to all measurements was 11% [55], and comparable rates (7–11%) have been reported by other authors [56,57,58].

Comorbidities. The following comorbidities/cofactors were identified in the analyzed group of patients with moderate to severe anaphylaxis: CVD > upper respiratory tract disease > lower respiratory tract disease > atopy > thyroid disease > diabetes mellitus type2 > infections > urticaria > gastrointestinal disease > exercise > osteoarthritis > neurological disease > alcohol.

Literature data indicate that in patients with anaphylaxis, the presence of cardiovascular disease has been shown to predispose them to fatalities [13,19,20,23,37], and it is probable that other chronic conditions such as renal and pulmonary problems would do likewise [59]. Concomitant cardiac conditions were an important predictor of severe anaphylaxis in the analysis of food-elicited reactions [35] and in patients with anaphylaxis induced by Hymenoptera venom [47,60], which is consistent with findings from our analysis. Recent medical history in elderly patients consisted of significantly more frequent cardiovascular, thyroid, and malignant diseases [61]. The above-mentioned studies suggest that comorbidities alone are regarded as a risk factor for severe anaphylaxis, although some researchers emphasized the significant effect of medications used [23]. Similar to our findings, cardiovascular diseases and asthma were reported as risk factors for severe anaphylaxis [54]. 

Asthma is associated with an increased incidence of anaphylaxis [51]. Contrasting data were presented by Worm et al. [35], who found no such association and even indicated that asthmatic patients had a lower risk of developing serious anaphylaxis (odds ratio (OR): 0.75, confidence interval (CI): 0.61–0.88).

Atopy was the fourth most common comorbidity in the analyzed group of patients with moderate to severe anaphylaxis. It was identified in 17% of cases, which is consistent with data reported by Versluis et al. [62] and Aurich et al. [60]. According to other researchers, atopic disease is identified in as many as 20–39% of patients with anaphylaxis [48,63,64,65]. It is clear that atopy increases the risk of systemic reactions because patients with atopy are at risk for food allergy, but they also appear to be at risk for events in general [59]. Similar conclusions were reached in a study on a population of beekeepers in Turkey and patients with exercise-induced or latex-induced anaphylaxis [54].

Thyroid diseases were identified in 30/382 patients and were the fifth most common comorbidity in the analyzed group. Perhaps this reflects the observed general increase in the incidence of thyroid diseases in the general Polish population. Nevertheless, data presented in the European anaphylaxis registry also indicate coexisting thyroid diseases in anaphylaxis [35] as a risk factor for moderate anaphylaxis (with an incidence rate of 1.5), and similar data were reported from the United States [20].

Infection—active infection was recorded as a cofactor in 19/382 cases of anaphylaxis (ca. 5%). Literature data indicate the potential role of infection in 1.3% to 11% [31], and even 29.8% of anaphylaxis cases [48]. On the other hand, in 257 cases (3.2%) recorded in the European Anaphylaxis Registry, physicians reported an active infection concomitant to anaphylaxis (e.g., upper respiratory tract infection or common cold) [35]. 

Menstruation, according to the available literature, is a cofactor of anaphylaxis in 8% to 12.1% of cases [31]. In our study, we did not find such a high rate, perhaps because patients could not remember details other than the symptoms of anaphylaxis. We recorded only one case in 236 women where menstruation was a cofactor. Nevertheless, single cases have been reported, indicating beyond any doubt, a significant effect of menstruation on the onset of anaphylaxis [66].

Considering modifiable extrinsic factors/cofactors of anaphylaxis, exercise was the most common one. In the analyzed material, this cofactor was identified in 11/382 cases. The second most common modifiable cofactor was alcohol, and it was identified in 9/382 cases. Awareness of the effect of these cofactors on the onset of anaphylaxis is important since they can be easily eliminated.

Exercise was identified as a cofactor in 2.9% cases, and, similar to a study by Oropeza et al. [48], FDEIA was diagnosed in five of these cases. An analysis conducted by Wölbing et al. revealed that exercise was a cofactor in 0% to 20.4% of anaphylaxis cases [31], which is consistent with our findings. The mechanism of action of this cofactor in anaphylaxis is explained by the activation of tissue transglutaminase (tTG), which results in the formation of large complexes of omega 5 gliadin and tTG. In addition, exercise increases the intestinal absorption of allergens and hence the concentration of these substances in the blood [67,68,69]. Christensen et al. concluded that exercise lowers the threshold and increases the severity of the reaction to the food [70].

Alcohol—In the analyzed registry, alcohol was a cofactor in 2.36% cases, while according to other researchers, it was involved in 1–15.2% [31,48,60], and even in up to 15% of cases of anaphylactic reaction according to some series [62,71]. It is also assumed that alcohol increases the gastrointestinal absorption of allergens [63], and induces modification in the expression of the tight junction-associated proteins ZO-1 and claudin-1 of the intestinal epithelium, thereby augmenting the permeability of the intestinal epithelial barrier [13]. According to the literature, only alcohol consumption could be implicated as a cofactor [72].

It is worth mentioning the importance of molecular diagnostics in allergology. Molecular tests help identify the cause of anaphylaxis, especially in patients with idiopathic anaphylaxis. In our analysis, the initial incidence rate of idiopathic anaphylaxis was 3.9% (15 patients), but according to literature data, it may be up to 20% [19]. Tests with new molecules for the determination of sIgE allowed for the identification of the direct cause of anaphylaxis in five patients, which accounts for 33% of cases with the established cause and initially labeled as idiopathic anaphylaxis. This rate is comparable to rates reported by other researchers [73], where the actual cause was identified in 45% of the previously unrecognized sensitizations. Moreover, recognized sensitization to heat-resistant molecules, e.g., lipid transfer proteins (LTP), has been reported as a predictive factor for severe anaphylactic reactions in the future [51,74,75]. Importantly, people with a diagnosed LTP allergy appear to be more likely to have a reaction to foods when a cofactor is present [76,77].

Hymenoptera-previous stings. In our analysis, 38% of patients with moderate to severe anaphylaxis following Hymenoptera stings previously had a generalized reaction to stings. Therefore, a history of generalized reaction is a significant risk factor for another anaphylaxis episode, compared to a previous severe local reaction (*p* < 0.001). Similar data have been reported since 1988 and have been described in a Hymenoptera venom study [52,78,79]. Even though 15% of patients with moderate to severe anaphylaxis had prior large local reactions to stings, similar to observations by Bilo et al. [60], venom immunotherapy is not recommended for large local reactions in either children [80,81] or adults [82]. Other factors may influence the decision to initiate VIT. These include occupations and/or hobbies where the risk of exposure is high, the culprit insect itself, concomitant cardiovascular diseases, other pathologies, or psychological factors arising from anxiety, which can seriously impair patient quality of life [83]. The natural history of large local reactions to Hymenoptera stings allowed the estimation of the risk of developing a systemic reaction after an initial large local reaction in about 4% of patients [84]; according to other authors, it is 2–15% [82,85,86]. Severino observed that in patients who had a history of a large local reaction, 24% did not experience any reactions, 52% reported a second large local reaction, and 24% had systemic reactions [85].On the other hand, concerning VIT, both American and European guidelines advise that it could be an acceptable option or recommended, in recurrent and troublesome large local reaction (LLR), to reduce the duration and size of future LLR, but only in special circumstances (i.e., frequent exposure, lifestyle factors) and after evaluating the cost/benefit profile [44,86,87,88,89].

Drugs-prior anaphylaxis. In the analyzed population, about 35% of patients with moderate to severe drug-induced anaphylaxis had a previous anaphylactic reaction to the same drug or a drug from the same chemical class, which is consistent with other reports [90,91]. This proves either low awareness among patients/doctors or the fact that patients did not try to explain previous health problems, which is consistent with observations made for a Polish population [49]. The risk factors for drug anaphylaxis are previous cardiovascular morbidity and older age [92]. The female predisposition to drug allergy can be explained by higher drug consumption, genetic factors, epigenetic changes, and discrepant hormonal interactions with immune cells [93]. However, literature data indicate that patients with a previous reaction, when re-exposed to the same drug, have a 21–60% risk of an immediate repeat reaction [94,95,96]. Among other conditions, atopy was reported as a risk factor for both NSAIDs, and antibiotic allergies [97,98], Kurt et al. [99] found that female sex, asthma, allergic rhinitis, and eczema diagnoses were associated with drug hypersensitivity reactions. According to a study based on data from the European Anaphylaxis Registry, 28% of elderly patients reported a previous allergic reaction to the same elicitor [60], which again substantiates the need for educating patients, people from their close environment, and healthcare professionals. Even though the first episode of anaphylaxis is unpredictable, further episodes in the same patient are preventable, but still happen [7,21].

### 5.1. Additional Material

The analysis of moderate and severe anaphylaxis cases was performed, excluding the youngest patients (0–18 years of age) *n* = 332, owing to the underrepresented children group (*n* = 50), which is emphasized in the Limitations section. Similar numerical values were obtained, which did not change the final conclusions of the work. 

Conclusions after excluding the children group:

Anaphylaxis occurred significantly more often in the age range 41–60; the only significant differences have been observed in women aged 19–40 vs. >60 and in women aged 41–60 vs. >60 as well as in men in the respective age ranges, people aged 19–60 constituted 86% of all the patients, women experienced anaphylaxis significantly more often, the most common comorbidities present in the analyzed group were: CVD, rhinitis (mainly allergic), bronchial asthma, atopy, thyroid diseases, diabetes (mainly type 2). When comparing the proportion of patients without comorbidities vs. one vs. two vs. three vs. four diseases/cofactors, a significant difference (*p* < 0.05) between patients with one cofactor vs. patients with three cofactors was observed. Analyzing the occurrence of anaphylaxis in people without any comorbidities/cofactors vs. people with at least one disease/cofactor, significant differences (*p* < 0.01) for each cause of anaphylaxis (drugs, food, insects) was observed. Drug-induced anaphylaxis recurred in about 37% of patients after contact with the same drug or a drug from the same group. Anaphylaxis after Hymenoptera stings occurred significantly more often when a patient had already had a similar anaphylaxis episode, as compared with a past local reaction.

### 5.2. Limitations

The population of the youngest children is underrepresented in the analyzed registry since our Allergy Clinic is a reference center for children older than five years and adults. Because this was a retrospective study, it may have been influenced by selection bias, and patients may not have remembered certain facts related to anaphylaxis. Not all patients completed all the investigations. Lack of detailed data on the history of anaphylaxis-cofactors was due to self-reported data. Challenge tests were performed only in a few cases, and the number of tests to measure tryptase levels were low. 

The analysis was not conducted for individual grades of anaphylaxis severity but for a pooled dataset of patients with moderate to severe anaphylaxis (grades II–IV). We did not assess the effect of drugs used by patients on the onset of anaphylaxis since no drug-related data were gathered. Another limitation is the lack of a corresponding control group. Therefore, we cannot draw inferences on which factors increase the risk of developing anaphylactic responses in the general population.

## 6. Conclusions

Anaphylaxis is an acute, severe, and life-threatening reaction. Cofactors were reported more frequently in patients with moderate to severe anaphylaxis, and it is important to take these factors into consideration when evaluating patients with anaphylaxis, as the cofactors may play a role in future anaphylactic reactions.

The presence of cofactors may explain why the intake of some foods sometimes leads to anaphylaxis, while in other cases, the same allergen induces a milder reaction or is even tolerated. It is necessary to refer every patient, after anaphylaxis, to an allergist for diagnosis.

Patients do not appear adequately equipped to deal with future episodes, which indicates the need for public health initiatives to improve anaphylaxis recognition, treatment, and prophylaxis.

## Figures and Tables

**Table 1:** Definitions of anaphylaxis.

WAO [3]	A serious life-threatening generalized or systemic hypersensitivity reaction. A serious allergic reaction that is rapid in onset and might cause death
EAACI [4]	A severe life-threatening generalized or systemic hypersensitivity reaction. An acute, potentially fatal, multi-organ system, allergic reaction.
AAAAI/ACAAI [5]	An acute life-threatening systemic reaction with varied mechanisms, clinical presentations, and severity that results from the sudden release of mediators from mast cells and basophils.
ASCIA [6]	Anaphylaxis is a serious, rapid-onset, allergic reaction that may cause death. Severe anaphylaxis is characterized by life-threatening upper airway obstruction, bronchospasm and/or hypotension.

**Table 2:** Classification of anaphylaxis severity [14].

Classification by Ring and Messmer	
Grade I	Generalized skin symptoms (e.g., flush, generalized urticaria, angioedema)
Grade II	Mild to moderate pulmonary, cardiovascular, and/or gastrointestinal symptoms
Grade III	Anaphylactic shock, loss of consciousness
Grade IV	Cardiac arrest, apnea

**Table 3:** The proportion of patients with an elevated basal tryptase level according to age ranges.

Age Range	*n* Patients	% of Whole Group *n* = 382	% of Tested *n* = 148	% of Age Group
0–18	0	0	0	0
19–40	1	0.26	0.68	0.79
41–60	11	2.88	7.43	6.88
>60	3	0.79	2.03	6.67
Total	15	3.93	10.14	3.93

**Table 4:** Characteristics of patients with Food-dependent exercise-induced anaphylaxis (FDEIA).

Allergen	Cofactor	Patient Age	Recurrence
Omega 5 gliadin	Exercise	24 years	8×
Omega 5 gliadin	Exercise + asthma	28 years	5×
Omega 5 gliadin	Exercise + asthma + alcohol	28 years	4×
Omega 5 gliadin	Exercise	27 years	2×
LPT	Exercise + asthma	31 years	4×

## Data Availability

The data presented in this study are openly available in [repository name e.g., FigShare] at [DOI:10.3390/ijerph17082787], reference number [42].

## Appendix A

Supplemental material with a simplified Network for Online-Registration of Anaphylaxis (NORA) questionnaire- from article” Clinical Manifestations and Causes of Anaphylaxis. Analysis of 382 Cases from the Anaphylaxis Registry in West Pomerania Province in Poland”.

The basis questionnaire—simplified version of the Network for Online-Registration of Anaphylaxis survey (NORA) from Berlin.

1. Did Your Patients, after contact with any factor, or spontaneously experience: difficulty breathing, wheezing, hypotension, cramping abdominal pain, diarrhea, vomiting, loss of consciousness? If so, complete the data
2. Year of the patient’ birth
3. Date of anaphylaxis onset
4. Place of reaction
5. Gender
6. Mark the organ systems involved
  6.1. Cutaneous: angioedema, flush, generalized erythema, generalized itching, generalized urticaria
  6.2. Respiratory: apnoea, dyspnoea, stridor
  6.3. Gastrointestinal: abdominal pain, diarrhoea, nausea, vomiting, incontinence
  6.4. Cardiovascular: loss of consciousness, drop in blood pressure, collapse, cardiac arrest, dizziness, tachycardia, disorientation
7. Mark the diagnostic tests used
  Medical Interview, skin tests, sIgE, tryptase, provocation, other
8. Was the reaction the first time?
9. Is the trigger factor known?—is the identified trigger of anaphylaxis?
  Is this medicine? substance
  Is this food? which one
  Is this venom? which one
  Other
10. Prevention
11. Please write other important details about this episode
